# Intraoperative Neuromonitoring: Evaluating the Role of Continuous IONM and IONM Techniques for Emerging Surgical and Percutaneous Procedures

**DOI:** 10.3389/fendo.2022.823117

**Published:** 2022-02-22

**Authors:** Pia Pace-Asciak, Jonathon O. Russell, Vaninder K. Dhillon

**Affiliations:** ^1^ Department of Otolaryngology – Head and Neck Surgery, University of Toronto, Toronto, ON, Canada; ^2^ Division of Endocrine Head and Neck Surgery, Johns Hopkins Otolaryngology-Head and Neck Surgery, Johns Hopkins Hospital, Baltimore, MD, United States

**Keywords:** intermittent intraoperative nerve monitoring, continuous intraoperative nerve monitoring, thyroid surgery, percutaneous, transcervical, scarless

## Abstract

Continuous and intermittent intraoperative nerve monitoring (IONM) has become an important asset for endocrine surgeons over the past few decades. The ability to determine neurophysiologic integrity of the recurrent laryngeal nerve (RLN) and external branch of the superior laryngeal nerve (EBSLN) on top of identification and meticulous dissection of the nerve in the surgical field, has allowed for advances in technology and improved outcomes when it comes to prevention of vocal fold immobility. This article aims to compare in review continuous and intermittent nerve monitoring (CIONM, IIONM), as well as review the current paradigms of their use. This article will also discuss the future of intraoperative nerve monitoring technologies in scarless thyroid surgery and percutaneous approaches to thyroid pathology in form of radiofrequency ablation (RFA).

## Introduction

The largest complication, and one with the most medio-legal implications during thyroid surgery, is injury to the recurrent laryngeal nerve (RLN) and external branch of the superior laryngeal nerve (EBSLN), during thyroid and parathyroid surgery. The laryngeal dysfunction that can result in problems to voice, swallowing and breathing and thus reduced quality of life (1) has made the role of intraoperative nerve monitoring (IONM) important for guiding favourable outcomes. Lahey’s observation in 1938 (2) that clear visualization of the RLN during thyroid surgery significantly lowers the incidence of nerve injury compared to surgery without visualization, is still the standard of care today (3–7). Even with visualization, advanced malignancy, re-operative surgery or aberrant anatomy can pose challenges making the nerve more difficult to isolate safely. While the role of IONM is still controversial, the technology provides added assurance of nerve integrity and planning, particularly for difficult cases and in the prevention of type I and type 2 injuries which may occur to the recurrent laryngeal nerve. Traction related injury to the nerve due to transection, clamping, ligation, pinching or bipolar coagulation can give rise to a segmental type 1 injury compared with a global loss of signal type 2 injury (8). As a result, the uptake of the technology amongst surgeons has resulted in an increase in its presence published in the literature, early adoption by younger surgeons and universal use in many parts of the world (9–12).

The use of IIONM and CIONM has been established with the International Neural Monitoring Study Group (INMSG), a multidisciplinary international group of surgeons and researchers, who published a comprehensive guideline on the technology (13). Both of these intraoperative nerve monitoring techniques are now being evaluated in a new arena, one that concerns the evolution of surgical procedures surrounding thyroid and parathyroid disease, from transcervical to endoscopic and robotic surgical approaches. The evolution of radiofrequency ablation and directed percutaneous approach to thyroid nodules also creates a new arena for nerve monitoring. This article aims to explore the role of IIONM and CIONM and their adaptability to the evolving world of minimally invasive and percutaneous approaches to the thyroid and parathyroid glands.

## A Comparison of the Technology

There are a number of existing techniques for nerve monitoring, some of which are still in development. The two most prevalent types which will be discussed in this paper include IIONM and CIONM.

The literature on the benefit of intraoperative nerve monitoring has evolved as the technology has advanced from IIONM to CIONM. Both of these techniques employ recording electromyographic signals from contraction of the vocal fold adductor muscles in response to stimulation of the RLN or vagus nerves using surface electrodes attached to an endotracheal tube (EMG-ETT). However, IIONM involves direct stimulation of the vagus or RLN with a handheld monopolar, whereas CIONM measures RLN functional integrity in real-time with a clip electrode mounted on the vagus nerve that continuously detects changes in amplitude in the electrophysiologic signal (8, 14). Thus, IIONM measures the intraoperative RLN signals in an isolated period of time, whereas CIONM can identify an impending traction related injury in real time allowing the surgeon to address concerns immediately to minimize injury.

The literature on IIONM has been conflicting regarding whether it decreases transient and permanent RLN injury (15–19). The literature shows conflicting results where some papers demonstrate that IIONM is beneficial for decreasing both transient and permanent RLN injury (16, 19), others show only transient RLN injury (15, 18), or only permanent RLN injury (17). Whereas, several studies have found that IIONM does not significantly reduce transient or permanent RLN injury (20, 23, 24). In a Cochrane review of five randomized controlled trials (RCT) with 1558 participants (781 patients were randomly assigned to IONM and 777 patients for visualization of the nerve only) found no conclusive evidence for the superiority or inferiority of IIONM over visualization of the RLN for permanent or temporary RLN paralysis, side effects or the time to complete the surgery (24).

CIONM in experienced hands can diminish permanent vocal fold palsy rates to 0% (1,314 nerves at risk), comparing favorably against 0.4% with IIONM (965 nerves at risk) (8, 25). The theory that CIONM allows real-time monitoring and may allow the surgeon to take remedial actions before the occurrence of a neurophysiologic injury, supports the use of CIONM in cases where the field is scarred and the surgical planes are obliterated, the presence of thyroid tumor that is infiltrating the tracheoesophageal groove, or other instances where there is a distortion of the normal anatomy (8).

One of the most common places for RLN is found to take place near or at the ligament of Berry (26). In a large multicenter trial, patients with persistent intraoperative loss of signal (115 nerves) were most often associated with traction type injury (83%), with 60% occurring in the ligament of Berry region (26). The ability to detect gradual EMG waveform decline with continuous nerve monitoring may allow for real time detection of an impending vocal fold paralysis, and may allow the surgeon to take remedial action, such as releasing the tethering of the thyroid to allow regain of nerve amplitude and function.

Studies directly comparing IIONM to CIONM are growing in number but still remain limited. One large observation study (21) found that rates of permanent but not transient vocal fold paralysis were improved with CIONM over IIONM. Of the 1526 patients that underwent thyroid surgery (788 using CIONM and 738 had IIONM), no permanent vocal fold palsy occurred with CIONM and four unilateral permanent vocal fold palsies (0.4%) were diagnosed after IIONM (P=0.019) (21). The study however was flawed by its lack of breakdown of the technology by individual surgeon as well as potential cognitive bias of CIONM use over IIONM. Other studies have found improved rates of transient RLN palsy with CIONM but not permanent, while still others have found no difference between modalities (22, 24, 27–34). A more recent comparative study of IIONM and CIONM in patients with benign and malignant thyroid disease over a decade demonstrated that in 6029 patients, the use of CIONM independently reduced the risk of early postoperative vocal cord palsy 1.8‐fold (odds ratio (OR) 0.56) and permanent palsy 29·4‐fold (OR 0.03) compared with IIONM. While visual nerve identification and IONM were used simultaneously, the study demonstrated standardization of nerve electrophysiological measurements along with pre and post-operative laryngeal exams (35, 36). This study provided the more robust advantage of CIONM over IONM in demonstrating higher sensitivity, specificity, positive predictive and negative predictive value.

Other factors that require more evaluation between IIONM and CIONM include cost, complexity and feasibility of use. CIONM is more costly and not as universal on the market as IIONM. The influence of surgical training on use of nerve monitoring appears to be favoring the use of the technology, as 83-93% of surveyed AHNS and International Endocrine Surgeons use IONM (10). While CIONM appears to have a higher advantage over IIONM in terms of results, the percentage of surgeons employing one technology over the other has not been identified. Possible complications associated with the use of the technology have been outlined by the INMSG, with more complications associated with CIONM for reasons listed above (13, 36).

## Preservation of EBSLN With Nerve Monitoring

The rates for nerve injury are likely quoted to be much lower than expected when the external branch of the superior laryngeal nerve is considered. This branch is a small peripheral branch that controls the cricothyroid musculature and allows for strong pitch and volume. The actual incidence of injury remains unknown by has been reported to be as high as 58% (37, 38) which makes this branch an important one during surgical dissection. Furthermore, the INMSG published guidelines on EBSLN preservation and nerve monitoring standards (39) which suggest that identification and monitoring of this branch intraoperatively is useful in preventing injury to the nerve. Furthermore, a more recent study on the routine identification and stimulation of the EBSLN using intermittent intraoperative nerve monitoring, performed before any dissection to the superior pole, leads to much higher rate of nerve conservation (40). There are currently on studies reviewing EBSLN monitoring in remote access thyroid surgery or RFA.

## Intraoperative Nerve Monitoring Amongst Newer Surgical Approaches

The evolution of surgical approaches to the thyroid and parathyroid glands is creating a landscape where nerve monitoring must adapt. While traditional transcervical approaches to the thyroid and parathyroid glands are still the norm, techniques that include endoscopic and robotic access are requiring a new design of monitoring technology. Endoscopic thyroid surgery, or scarless thyroid surgery, has taken off in the past decade, and is become a fast approaching means by which many patients opt to have surgery in the neck (41–45). This is similar to robotic approaches to the thyroid gland, whether they be from the axilla, breast line and/or intraorally (46–48). These approaches require identifying the nerve from a different angle or view from the traditional approach.

The literature on intraoperative nerve monitoring in scarless thyroid surgery has not been well defined. However, animal studies evaluating the role of CIONM in transoral thyroidectomy have determined its feasibility (49).

In a retrospective review, Russell et al, looked at the outcomes in 200 patients treated with the transoral endoscopic thyroidectomy vestibular approach (TOETVA) and compared to 333 patients treated by the conventional transcervical approach thyroidectomy (TCA) (41). In both treatment arms, IIONM was used and no statistical difference was found in the rate of temporary RLN injury (4.5% vs. 2.1%, p=0.124) thus demonstrating the safety of this approach. Dissection of the recurrent laryngeal nerve by TOETVA is not as extensive as it is in transcervical approaches and provides a bird’s eye view of the RLN, thereby requiring greater attention and precision in monitoring the RLN for IIONM, particularly for the novice surgeon looking to expand their surgical armamentarium. The handheld probes are being transformed into those with adaptability to endoscopic and robotic arms, in order to reach the nerve during the case. On the other hand, new methods to tag the vagus nerve for CIONM, in a surgical field where the vagus is not circumferentially accessible need to be developed.

Minimally invasive techniques such as radiofrequency ablation (RFA), currently are the next frontier for endocrine surgery. RFA is a thermally ablative method that targets nodules of interest in real time, with ultrasound guidance rather than with an incision by the traditional open neck approach. Minimally invasive techniques pose new challenges when it comes to nerve monitoring given that the patient is awake versus intubated with a neutrally monitored endotracheal tube. With such cases, sonography is used to monitor the tip of the electrode probe during ablation to ensure that the tip does not extend beyond the posterior or lateral capsule of the nodule into the ‘danger triangle’ where the recurrent laryngeal nerve sits (50).

A study of 16 patients from Xiamen China underwent radiofrequency ablation with the use of neural monitoring of the larynx using a pair of cutaneous surface electrodes attached to the neck at the thyroid cartilages as well as grounding electrodes to the upper arm. The nerve stimulation needle was connected to a saline syringe and used as a neural probe after hydrodissection of the thyroid capsule from the surrounding tissue. Fourteen of the sixteen patients tolerated the procedure with local anesthesia and two had to be put under sedation. The technique described and documented results are similar to IIONM. Complications arising from the nerval monitoring included pain, local hematoma, vasovagal reaction bradycardia, hypertension and vomiting. 100% of EMG signals were obtained demonstrating feasibility of IIONM during RFA (51).

In another prospective series of thirteen nodules treated with RFA, Sinclair et al, used continuous intraoperative neuromonitoring while ablating nodules localized in the posterior thyroid capsule near the danger triangle (52). The technology used for continuous nerve monitoring involved an EMG-ETT and eliciting the laryngeal adductor reflex and continuous monitoring of RLN and vagal nerve function (14, 53, 54). Up to 40 W of power was utilized while adjacent to the posterior capsule and no evidence of amplitude changes were noted, nor was there a change in pre- or postoperative laryngoscopy and voice assessments (52). CIONM can be a useful adjunct for ensuring safe and thorough treatment when ablating near the danger triangle, however all patients were intubated.

The shift in paradigm to ‘less is more’ is something being witnessed in thyroid and parathyroid surgery. The data associated with how the technology is utilized and capable of preserving neural function is not robust, beyond being evaluated in a few case series. The trajectory for IIONM and CIONM is in the advent of remote access and percutaneous monitoring devices which can help the surgeon focally approach the thyroid and parathyroid disease of interest while ensuring that the RLN and EBSLN are monitored for inadvertent injury. The manifestation of this type of technology is yet to be determined.

## Conclusion

The role of IIONM and CIONM in thyroid surgery has been established as a well-versed adjunct to determining the integrity of the recurrent laryngeal and the external branch of the superior laryngeal nerve during thyroid and parathyroid surgery. The next frontier is the evolution of these technologies within the confines of endoscopic and robotic surgery as well as radiofrequency ablation. Preliminary work suggests that there is an adaptability of the technology for these new approaches in endocrine surgery.

## Author Contributions

PAA contributed to the literature review, manuscript writing. VKD contributed to the literature review, manuscript writing and revision as well as manuscript finalization. All authors contributed to the article and approved the submitted version.

## Conflict of Interest

The authors declare that the research was conducted in the absence of any commercial or financial relationships that could be construed as a potential conflict of interest.

## Publisher’s Note

All claims expressed in this article are solely those of the authors and do not necessarily represent those of their affiliated organizations, or those of the publisher, the editors and the reviewers. Any product that may be evaluated in this article, or claim that may be made by its manufacturer, is not guaranteed or endorsed by the publisher.
